# fragHAR: towards *ab initio* quantum-crystallographic X-ray structure refinement for polypeptides and proteins

**DOI:** 10.1107/S2052252519015975

**Published:** 2020-01-17

**Authors:** Justin Bergmann, Max Davidson, Esko Oksanen, Ulf Ryde, Dylan Jayatilaka

**Affiliations:** a Department of Theoretical Chemistry, Chemical Center, Lund University, PO Box 124, SE-221 00 Lund, Sweden; b School of Molecular Sciences M310, University of Western Australia, 35 Stirling Highway, Crawley 6009, Australia; c Instruments Division, European Spallation Source ESS ERIC, PO Box 176, SE-221 00 Lund, Sweden

**Keywords:** Hirshfeld atom refinement, quantum crystallography, peptides, H atoms

## Abstract

The first *ab initio* aspherical structure refinement against experimental X-ray structure factors for polypeptides and proteins using a fragmentation approach to break up the protein into residues and solvent, thereby speeding up quantum-crystallographic Hirshfeld atom refinement (HAR) calculations, is described. It it found that the geometric and atomic displacement parameters from the new fragHAR method are essentially unchanged from a HAR on the complete unfragmented system when tested on dipeptides, tripeptides and hexapeptides. The largest changes are for the parameters describing H atoms involved in hydrogen-bond interactions, but it is shown that these discrepancies can be removed by including the interacting fragments as a single larger fragment in the fragmentation scheme. Significant speed-ups are observed for the larger systems. Using this approach, it is possible to perform a highly parallelized HAR in reasonable times for large systems. The method has been implemented in the *TONTO* software.

## Abstract 2

A method to fragment quantum-chemical calculations in Hirshfeld-atom refinement is reported, which allows accurate aspherical atom X-ray refinement of polypeptides and proteins.

## 1. Introduction   

In order to understand the function of proteins and to control or modify enzymatic reactions, for example using drugs or by mutation, it is important to know the detailed atomic structure. The most common way to obtain this kind of information is through X-ray diffraction of protein crystals. Unfortunately, H atoms are typically not discerned in protein crystal structures because they have only one electron and therefore scatter X-rays weakly. This is problematic because the H atoms determine the charge and protonation states of many molecules and residues, and they determine the direction of hydrogen bonds, which are crucial both for the structures of proteins and for the catalytic mechanisms of enzymes. Therefore, neutron single-crystal diffraction experiments are used as a gold standard to obtain hydrogen positions. Unfortunately, they are more expensive and time-consuming than X-ray crystallographic experiments and are sometimes even impossible because large crystals are needed.

At ultrahigh resolution (<1 Å), well-ordered H atoms start to be visible in crystal structures. However, protein crystals scarcely scatter to such a resolution: only 671 of the data sets (0.4%) in the PDB are in this resolution range. Moreover, such structures typically give *X*—H bond lengths that are systematically too short (by ∼0.12 Å). The reason for this is that most protein crystallographic refinement software employs the independent atom model (IAM) to obtain atom positions and displacement parameters. IAM uses a superposition of smeared *spherical* atomic densities to describe the averaged electron density in the periodic crystal (Coppens, 1997 ▸). However, the electron density of an H atom is not spherical and it is not centred on the nucleus. Instead, the maximum of the electron density in an *X*—H bond is shifted from the nuclei of the H atom into the bond. The atomic displacement parameters (ADPs) of the H atoms are even more difficult to obtain correctly in X-ray crystal structures. In fact, the positions of non-H atoms with lone pairs may also be shifted slightly, another example of a nonspherical electron density.

Fortunately, there are methods to obtain accurate H-atom positions and ADPs from X-ray diffraction data, based on an aspherical electron-density description of the atoms, but these are so far available only for small molecules with a resolution of <0.85 Å. Destro & Merati (1995 ▸) were the first to demonstrate that this is possible using the Hansen–Coppens multipole model and several others have used the same approach (Zhurov *et al.*, 2011 ▸; Zhurov & Pinkerton, 2013 ▸). Dittrich *et al.* (2005 ▸) showed that it is possible to obtain *X*—H bond lengths in agreement with those obtained from neutron structures using a database of aspherical atomic form factors fitted to structure factors obtained from quantum-mechanical (QM) calculations. Very recently, Malaspina *et al.* (2019 ▸) reported a quantum-mechanical database for chemical fragments that can be used to build a whole protein, which also recovers excellent *X*—H bond lengths.

Hirshfeld atom refinement (HAR) is a method that allows the determination of H-atom positions from standard-resolution small-molecule X-ray crystallography (Jayatilaka & Dittrich, 2008 ▸; Capelli *et al.*, 2014 ▸). In HAR, a wavefunction for the molecule in the crystal geometry is calculated. From this wavefunction an electron density (ED) is obtained, which is then partitioned into aspherical atomic pieces using Hirshfeld’s stockholder partitioning scheme. The aspherical atomic structure factors, *i.e.* the Fourier transform of the Hirshfeld atomic ED, are then calculated and used in a least-squares refinement against the experimental X-ray structure factors. HAR has the advantage that it does not require any aspherical form factors stored in databases or tables. Neither are the aspherical atomic form factors approximated using multipoles. Instead, they are calculated by QM methods when required. With HAR, H-atom positions can be obtained in quantitative agreement with neutron diffraction results even at 0.8 Å resolution (Woińska *et al.*, 2016 ▸). It should be emphasized that non-H-atom positions obtained from HAR are more precise than those that can be obtained from quantum-chemical energy optimizations, even with high-level methods.

Unfortunately, there is no free lunch: HAR is many orders of magnitude slower than IAM and database methods. In particular, the time consumption increases sharply with the size of the studied system, because calculating a QM wavefunction is very time-consuming for large molecules. This makes HAR currently unfeasible for large systems such as polypeptides and proteins.

Of course, the problem of performing QM calculations on large systems has occupied quantum chemists for a long time and many techniques have been developed, including methods that are linear-scaling in the number of atoms.

A well-established approach for modelling proteins is the QM/MM method, which describes a region of interest, for example the active site, using a QM method and the remainder using a molecular-mechanics (MM) model (Warshel & Levitt, 1976 ▸; Singh & Kollman, 1986 ▸; Senn & Thiel, 2009 ▸; Ryde, 2016 ▸). QM/MM methods can be used for the refinement of low- and medium-resolution protein structures, when combined with the joint X-ray/MM refinement method of Brünger *et al.* (1987 ▸). The result is the quantum-refinement method of Ryde *et al.* (2002 ▸). Merz and coworkers have suggested a similar method in which the complete protein is described by semiempirical quantum-mechanical calculations (Yu *et al.*, 2005 ▸). Importantly, this method has been integrated into the widely used *Phenix* protein structure-refinement program (Borbulevych *et al.*, 2014 ▸; Liebschner *et al.*, 2019 ▸). An alternative approach, *Q*|*R*, has also been suggested in which the full protein is treated by density-functional theory (Zheng *et al.*, 2017 ▸). All of these methods still make use of spherical atomic form factors.

Another way to speed up the QM calculations is to fragment the full system into smaller subsystems, for which the wavefunction is calculated, and then ‘piece’ the results together. This approach, which is obviously linear-scaling, makes QM calculations feasible for proteins (Stoll, 1992 ▸; Doll *et al.*, 1997 ▸; Zhang & Zhang, 2003 ▸; Söderhjelm & Ryde, 2009 ▸; Yang, 1991 ▸; Lee *et al.*, 1996 ▸; Yang & Lee, 1995 ▸; Kohn, 1996 ▸; Dixon & Merz, 1996 ▸; Gogonea *et al.*, 2000 ▸; Stewart, 1996 ▸; Daniels *et al.*, 1997 ▸; Daniels & Scuseria, 1999 ▸; Scuseria, 1999 ▸). These methods have been reviewed by Collins & Bettens (2015 ▸), and a general program to implement them by scripting other *ab initio* packages has been presented by Kobayashi *et al.* (2019 ▸).

All of these methods focus on obtaining the *energy*, whereas the ED, if it is produced at all, is just a byproduct. Walker & Mezey (1994 ▸) reported a Mulliken-like method to produce EDs for proteins from fragments, but it has not been used for X-ray structure refinement. Massa *et al.* (1995 ▸) proposed the kernel density method to obtain the ED of large systems and applied it to a cyclic hexapeptide whose structure was taken from X-ray measurements, but no X-ray structure refinement was attempted. Very recently, Northey & Kirrander (2019 ▸) developed a fragmentation approach for ED calculated by the *ab initio* X-ray diffraction method, fitted to X-ray free-electron laser data, and Malaspina *et al.* (2019 ▸) derived a database of extremely localized molecular orbitals for chemical fragments which can be used to build a protein and permit large HAR calculations, a method called HAR-ELMO.

In this paper, we develop a fragmentation approach to speed up the QM protein structure-factor calculations required for HAR with single-crystal data. It is based on the molecular fractionation with conjugate caps (MFCC) approach of Zhang & Zhang (2003 ▸). Our method, which we call fragHAR, is described in the next section and is tested on three oligopeptide systems for which high-quality X-ray diffraction data are available: a dipeptide, a tripeptide and a hexapeptide. The results are compared against full HAR calculations; the accuracy of HAR relative to neutron diffraction measurements has already been established (Capelli *et al.*, 2014 ▸; Fugel *et al.*, 2018 ▸).

## 2. Theory and methods   

### 2.1. Hirshfeld atom refinement   

The HAR calculations were performed as described in the literature (Jayatilaka & Dittrich, 2008 ▸; Capelli *et al.*, 2014 ▸; Woińska *et al.*, 2014 ▸). The atomic form factors were calculated numerically using Becke integration grids (Becke, 1988 ▸). The least-squares procedure is performed using standard methods, refining against the structure-factor magnitudes, and no attempt was made to parallellize this part of the code, because it does not limit the calculations for the small systems considered here. All calculations were performed with a development version of the *TONTO* software (Jayatilaka & Grimwood, 2003 ▸).

### 2.2. The fragHAR fragmentation scheme   

Zhang & Zhang (2003 ▸) introduced a method to achieve a linear scaling for the calculation of the QM energy of proteins, called molecular fractionation with conjugate caps. This method breaks a protein into residues by cutting the peptide bonds and replacing the *R*NH– group with CH_3_NH– and the *R*′C=O group with CH_3_C=O. Larger fragments may also be used (Antony & Grimme, 2012 ▸) but, as we show below, this procedure works well for X-ray structure refinement.

**Figure 1:**
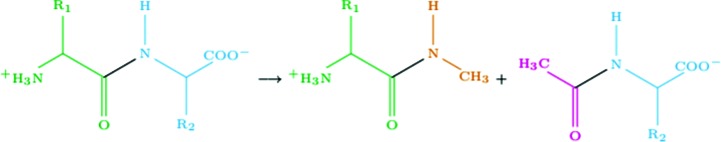
The molecular fractionation with conjugate caps (MFCC) procedure for cutting a dipeptide (left) across the peptide bond (shown in black), producing two fragment molecules (right), which are then ‘capped’ with –CH_3_C=O (red) and –NHCH_3_ (orange) groups, comprised of the neighbour and next-neighbour non-H atoms.

Truncating H atoms are placed in the direction of the actual atoms at standard distances (Allen & Bruno, 2010 ▸). The fragmentation scheme is illustrated schematically in Fig. 1 ▸ for a dipeptide. Solvent molecules are treated as separate fragments.

In our implementation, bonded atoms are defined according to the Cambridge Crystallographic Database criterion,
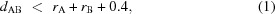
where *r*_A_ and *r*_B_ are the covalent radii of atoms A and B, respectively, and *d*_AB_ is the distance between them (all in Å). Using this criterion, hydrogen bonds are not taken into account, but such connections are easy to introduce by instead using van der Waals radii in (1). The assumption that only next-nearest neighbour non-H atoms are sufficient to provide a good model of the ED central fragment has already been established by Dittrich *et al.* (2002 ▸). This scheme is easily generalized, if required.

In our fragHAR approach, structure factors are calculated for the central (non-overlapping) part of each fragment (*i.e.* for each residue separately) using the wavefunction for each capped fragment. These structure factors are then directly employed in the standard HAR procedure, without any modification. Thus, there is no need to calculate structure factors for any conjugate capping groups.

An important difference concerning energy fragmentation methods versus electron-density fragmentation methods is that for X-ray structure refinement only the aspherical atomic structure factors are required. Therefore, there is no need to subtract the energies of the capping groups (Zhang & Zhang, 2003 ▸).

### 2.3. Parallelization and timing   

For large systems, the fragmentation scheme will of course speed up the aspherical atomic structure-factor calculations because the calculations are performed on smaller molecules; the time will be no more than *N*_frag_ times the calculation time for the largest fragment, *i.e.* linear scaling in the number of fragments *N*_frag_. Provided that the least-squares procedure is not a bottleneck, a fixed calculation time may be achieved if each of these fragment calculations is performed in parallel on separate processors. We have implemented such a parallelization using the MPI protocol, whereby the QM calculations on each fragment are distributed to free processors as soon as they become available.

### 2.4. Choice of model systems and experimental data   

**Figure 2:**
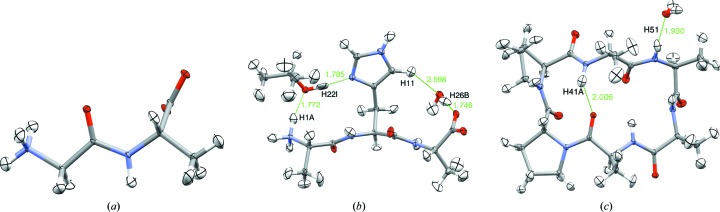
Crystal structures (100 K) of the three peptide model systems with 50% ADP probability ellipsoids. Hydrogen bonds are shown in green. (*a*) Gly-Ala (GA), (*b*) Ala-His-Ala (AHA), (*c*) *cyclo*-(Ala)_4_-(d,l-Pro)_2_ (A_4_P_2_).

Three published test systems (Fig. 2 ▸) were used to show that the fragmentation is a reasonable approximation to obtain a good refined structure. The systems were the dipeptide Gly-Ala (GA; Capelli *et al.*, 2014 ▸), the tripeptide Ala-His-Ala (AHA; with 2-propanol and water as solvent; Grabowsky *et al.*, 2009 ▸) and the hexapeptide *cyclo*-(Ala)_4_-(d,l-Pro)_2_ (A_4_P_2_; with one water molecule as solvent; Dittrich *et al.*, 2002 ▸). The solvent molecules were treated as separate fragments in fragHAR. As reference, a full HAR calculation with a single wavefunction for the complete structure was used.

### 2.5. Details of wavefunction calculations   

All QM calculations (both for HAR and fragHAR) were performed with Hartree–Fock wavefunctions using the cc-pVDZ basis of Dunning (1989 ▸). This has previously been found to be a proper level of theory when refining QM wavefunctions to structure factors, giving *X*—H bond lengths in agreement with neutron diffraction results (Capelli *et al.*, 2014 ▸; Fugel *et al.*, 2018 ▸). Note that the QM calculations are used to obtain the aspherical electron density (to calculate structure factors), not to optimize the geometries on a potential energy surface.

### 2.6. Quality statistics   

We use standard crystallographic statistics to compare data sets (in our case fragHAR and HAR refinement; Schwarzenbach *et al.*, 1995 ▸). In addition, we use the mean of the ratio of data pairs (〈*r*_fragHAR_/*r*_HAR_〉) and the mean absolute difference between the data pairs (〈|Δ*r*|〉 = 〈|*r*_fragHAR_ − *r*_HAR_|〉). To establish statistical agreement between two parameter sets {*A_i_*} and {*B_i_*} we use the weighted root-mean-square deviation (Capelli *et al.*, 2014 ▸; Schwarzenbach *et al.*, 1995 ▸),
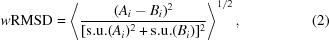
where s.u. is the standard uncertainty and values in the range 0 ≤ wRMSD < 1 indicate that the two data sets are in statistical agreement.

## 3. Results and discussion   

### 3.1. Comparison of goodness-of-fit parameters   

Table 1 ▸ summarizes the crystallographic data and the refinement results for both the fragHAR and the reference HAR calculations for the three tested oligopeptides. It can be seen that there are only negligible differences in both the residue density peaks (third decimal place) and the *R* values (second decimal place) between the two refinements.

### 3.2. Comparison of bond lengths   

**Figure 3:**
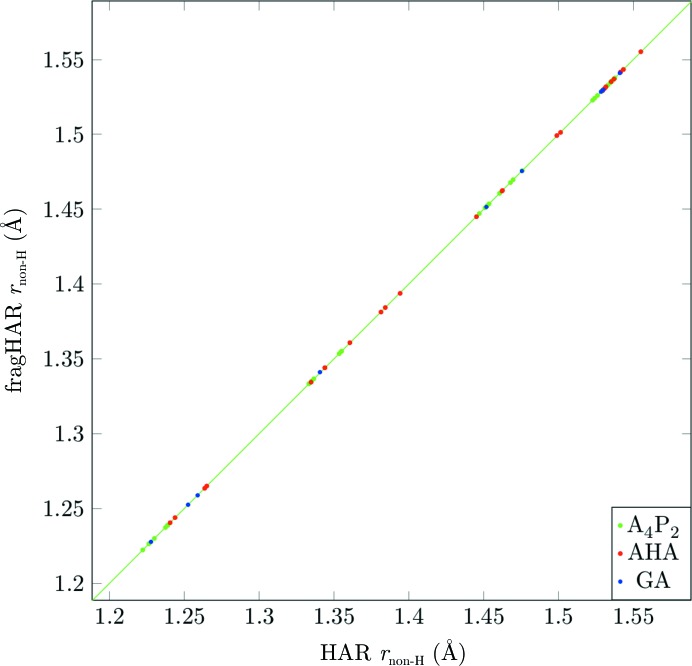
Bond lengths between non-H atoms from fragHAR calculations plotted against reference HAR values. Error bars are depicted, but are invisible to the eye on this scale.

Fig. 3 ▸ compares the bond lengths involving non-H atoms obtained by HAR and fragHAR for the three peptides. It can be seen that the two sets show a perfect agreement. Therefore, we do not present any deeper statistical analysis (more detailed graphs are provided in the supporting information). Clearly, fragHAR does not represent any significant approximation compared with HAR for the non-H atoms.

The results for the *X*—H bond lengths obtained in the refinements are shown in Table 2 ▸. The bond lengths are divided into three classes, C—H, N—H and O—H, in order to make the comparison more detailed. It can be seen that the C—H bond lengths from the two refinements are in statistical agreement (*w*RMSD = 0.2–0.8). There is a minimal tendency for the fragHAR bond lengths to be slightly shortened (〈*r*_fragHAR_/*r*_HAR_〉 = 0.998–0.999), but the deviation from unity is less than the standard uncertainty. The N—H bond lengths in GA are also in statistical agreement (*w*RMSD = 0.4), but for the larger oligopeptides there is a slight disagreement in the N—H bonds, with *w*RMSDs of 1.2 and 1.0. Likewise, the O—H bond lengths in the tripeptide AHA show a statistical disagreement, with *w*RMSD = 2.4, whereas the two O—H bonds in A_4_P_2_ agree between the two methods (*w*RMSD = 0.5).

**Figure 4:**
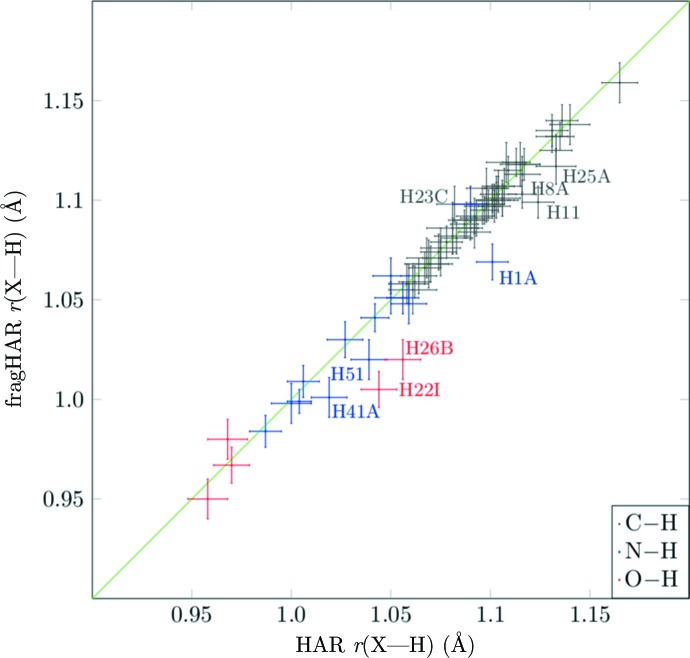
*X*—H bonds (with error bars) in all model compounds for fragHAR calculations versus reference HAR values. Bonds with notable differences are marked with the corresponding H-atom name.

To investigate the reason for these differences, we plotted the bond lengths from fragHAR and HAR in Fig. 4 ▸. It can be seen that for most bonds the results of the two methods agree, but there are are a few outliers that are identified by atom label (as shown in Fig. 2 ▸). It can be seen that all of the outliers are associated with H atoms involved in intermolecular hydrogen bonds between the residues or the solvent molecules. Such interactions are not modelled in the fragHAR method with residue fragments. It is remarkable that the X-ray data contain sufficient information to distinguish these small hydrogen-bonding effects via their neglect in the fragHAR model.

**Figure 5:**
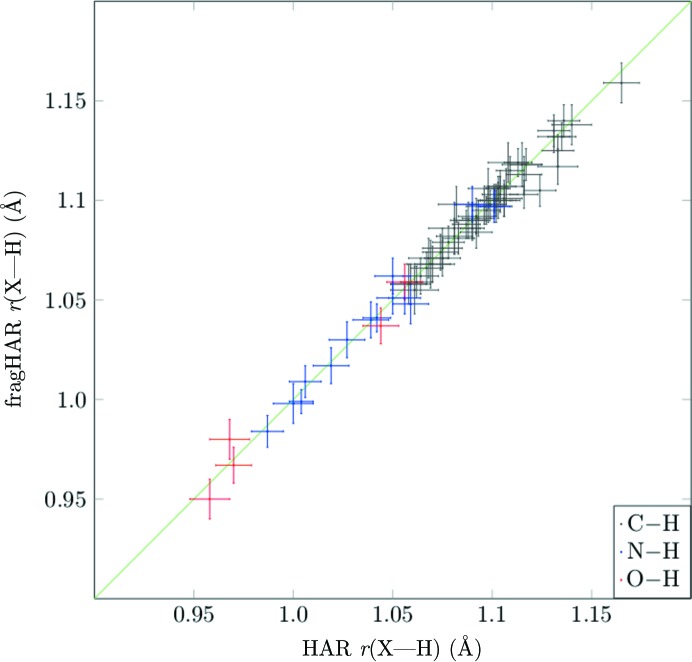
*X*—H bonds (with error bars) in all model compounds for fragHAR with fragments ‘joined’ across hydrogen bonds versus reference HAR calculations.

To determine whether this shortcoming may be eliminated, we joined the two fragments involved in the hydrogen bond of interest and treated them as a single fragment. Fig. 5 ▸ shows that such a procedure solves the problem for all *X*–H bond lengths. For example, the N–H41A bond length in A_4_P_2_ (H41A makes an intramolecular hydrogen bond, as can be seen in Fig. 2 ▸) improves from 1.000 (10) Å for standard fragHAR to 1.017 (9) Å with the doubled fragment, compared with 1.020 (9) Å for HAR. Therefore, these small discrepancies can be corrected if the size of the fragment is increased to include all of the residues that are hydrogen-bonded to it. As the calculations are performed in parallel, the time taken will still be roughly equal to the time for the largest fragment.

Finally, we note that both HAR and fragHAR give *X*—H bond lengths that are in agreement with those obtained by neutron crystallography, in contrast to IAM, which gives *X*—H bond lengths that are too short. This illustrates that X-ray data with a resolution lower than 0.8 Å can provide hydrogen positions that are as accurate as those from neutron crystallography (Jayatilaka & Dittrich, 2008 ▸; Woińska *et al.*, 2014 ▸; Capelli *et al.*, 2014 ▸; Fugel *et al.*, 2018 ▸).

### 3.3. Comparison of atomic displacement parameters   

Table 3 ▸ compares the ADPs obtained by fragHAR and HAR for non-H and H atoms. For the non-H atoms, the mean absolute differences between the ADPs from fragHAR and HAR are at least four orders of magnitude smaller than the *w*RMSDs and the mean ratios are 0.999–1.000. For the H atoms, the ratios between the ADPs for the two types of refinement are 1.00–1.01 and the *w*RMSD is in the range 0.2–0.6. Thus, the ADPs of the two methods are in statistical agreement.

**Figure 6:**
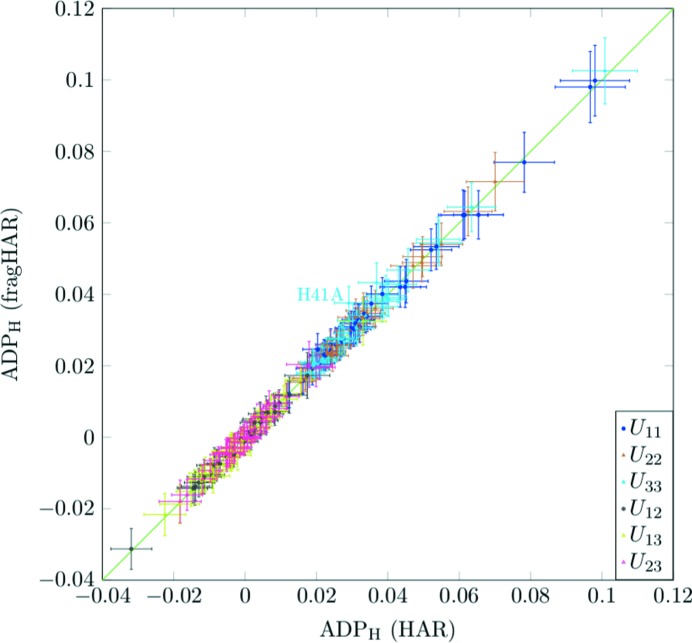
Hydrogen ADPs (with error bars) obtained with fragHAR versus those from HAR for the A_4_P_2_ system. The hydrogen-bonded H41A atom is labelled.

Although most ADPs from fragHAR and HAR refinements are in statistical agreement, if they are plotted against each other, as in Fig. 6 ▸, a few ADPs with significant deviations can be observed. Again, these outliers are for H atoms involved in hydrogen bonds to other fragments. The discrepancy also increases with the strength of the hydrogen bonds, so that there is a larger difference for short hydrogen bonds than for longer hydrogen bonds (further details are given in the supporting information). Again, it is interesting to see that the X-ray data contain sufficient information to distinguish these effects in the ADPs, and even give some indication of their magnitude. As seen for the bond lengths, the hydrogen ADPs are also improved if both fragments involved in hydrogen bonds are merged together in a single fragment (for example, 〈|Δ*U_ij_*|〉 for H41A decreases from 0.012 to 0.003).

### 3.4. Timing   

**Figure 7:**
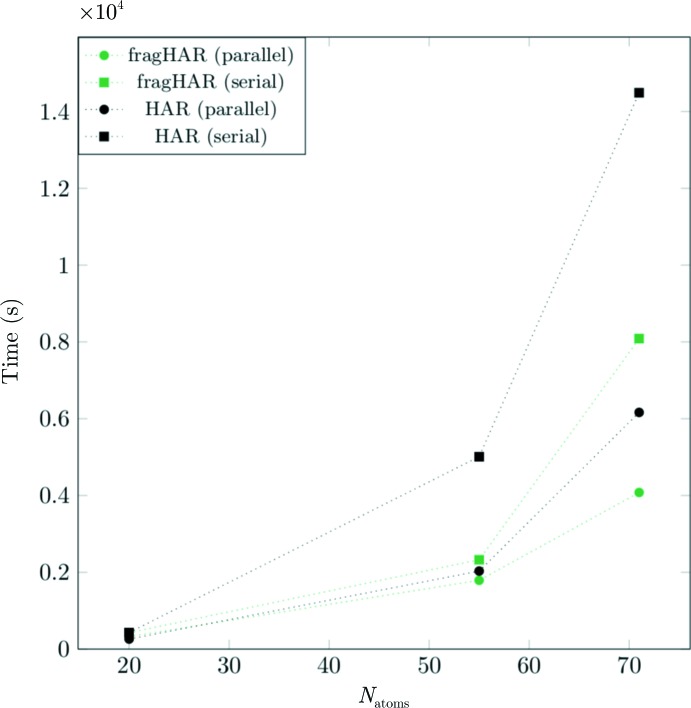
Timing of the fragHAR (green) and HAR calculations (black) for single-processor serial (square) and parallel (circles) calculations for GA (two processors), AHA (four processors) and A_4_P_2_ (four processors).

Fig. 7 ▸ shows the timings for HAR and fragHAR calculations for single-processor (serial) and parallel calculations. fragHAR gives a significant reduction in calculation time for the larger systems (*i.e.* those with more than two fragments) for the serial calculations. When using parallel calculations, there is no difference in time for the tripeptide because the time is determined by the largest fragment calculation. However, for the larger hexapeptide fragHAR takes approximately half the time compared with HAR. Of course, even larger speed-ups are expected for larger systems since if every fragment is assigned its own processor in a large parallel system the wall time required for any size of protein will be constant.

## 4. Conclusion   

We have described a method to improve the speed of Hirshfeld atom refinement (HAR) on peptides and proteins by breaking them up into capped residue fragments using the MFCC approach and performing wavefunction calculations on these fragments. Based on tests on three oligopeptide systems, we show that the new fragHAR method produces essentially the same *R* factors, bond lengths and ADPs as a full HAR calculation. Significant differences are only observed for H atoms involved in hydrogen bonds between the fragments. This problem can be fixed by enlarging the fragment to include the interacting group.

The fragHAR approach scales linearly with the size of the studied system, and with a sufficiently large parallel computer the calculations would take a fixed time, depending only on the length of the calculation on the largest fragment.

While ultrahigh-resolution data for proteins remain rare, there is a growing number of systems for which a resolution of <0.8 Å can be obtained. Therefore, the fragHAR method of quantum-crystallographic refinement, which avoid the use of restraints, could contribute to determining hydrogen positions in proteins.

Other serious problems remain to be solved. For example, it remains to be shown what resolution will be needed for the fragHAR method to give reliable H-atom positions in proteins, because the effects of disorder may swamp any H-atom signal. The treatment of disorder is also somewhat tricky. Here, the use of appropriate restraints and constraints to maintain a chemically reasonable model will be important: some parts of a protein will always be disordered no matter the quality of the data. Still, it should be noted that fragHAR provides a natural solution to groups with alternative configurations, since one could use a separate fragment for each conformation; by contrast, standard HAR would require separate calculations of the entire macromolecule for each alternative conformation.

However, methods to treat disordered solvent by flattening are well developed, as are methods to deal with constraints and restraints (Sheldrick, 2015 ▸). Also, we will shortly report an extension to HAR which treats disorder. With theses comments and the results of this paper in hand, there would seem to be good prospects for the use of fragHAR for proteins for which ultrahigh-resolution data can be obtained.

## Supplementary Material

Details of refinement results for each model compound; indicative parallel program timings. DOI: 10.1107/S2052252519015975/fc5039sup1.pdf

## Figures and Tables

**Table 1:** Crystallographic refinement details for fragHAR versus HAR obtained using Hartree–Fock wavefunctions with the cc-pVDZ basis set

	GA		AHA		A_4_P_2_	
	fragHAR	HAR	fragHAR	HAR	fragHAR	HAR
Formula	C_5_H_10_N_2_O_3_		C_15_H_29_N_5_O_6_		C_22_H_36_N_6_O_7_	
System	Orthorhombic		Monoclinic		Orthorhombic	
Space group	*P*2_1_2_1_2_1_		*P*2_1_		*P*2_1_2_1_2_1_	
Wavelength (Å)	0.5259		0.560		0.5583	
*a* (Å)	7.472 (2)		8.7410 (17)		10.1280 (10)	
*b* (Å)	9.4907 (6)		9.4200 (19)		12.4860 (10)	
*c* (Å)	9.7169 (8)		11.989 (2)		9.5070 (10)	
α = γ (°)	90		90		90	
β (°)	90		95.49 (3)		90	
*T* (K)	100 (2)		100 (2)		100 (2)	
*d* (Å)	0.65		0.43		0.38	
*N*_meas_	2431		12261		22268	
*N*_atoms_	20		55		71	
*N*_fragments_	2	1	5	1	7	1
ρ_max_ (e Å^−3^)	0.1487	0.469	0.1573	0.1606	0.2423	0.2400
ρ_min_ (e Å^−3^)	−0.1579	−0.1792	−0.2000	−0.1996	−0.1988	−0.1967
*R*(*F*) (%)	1.70	1.82	2.41	2.39	3.29	3.29
*wR*(*F*) (%)	1.45	1.55	2.10	2.09	2.88	2.89

**Table 2:** Comparison of *X*—H bond lengths obtained with fragHAR and HAR Values in parentheses represent the sample standard deviations.

Compound	Bond	*N*_data_	〈*r*(*X*—H)〉, fragHAR (Å)	〈*r*(*X*—H)〉, HAR (Å)	〈*r*_fragHAR_/*r*_HAR_〉	〈|Δ*r*|〉	*w*RMSD
GA	C—H	6	1.09 (2)	1.09 (2)	0.999 (4)	0.003 (4)	0.40
	N—H	4	1.03 (2)	1.03 (2)	0.999 (3)	0.002 (3)	0.32
AHA	C—H	20	1.11 (2)	1.11 (2)	0.998 (9)	0.007 (8)	0.8
	N—H	6	1.05 (4)	1.06 (4)	0.99 (2)	0.01 (2)	1.24
	O—H	3	1.00 (4)	1.02 (4)	0.98 (3)	0.02 (3)	2.4
A_4_P_2_	C—H	30	1.08 (3)	1.08 (3)	0.999 (3)	0.001 (2)	0.18
	N—H	4	1.00 (2)	1.01 (2)	0.989 (8)	0.01 (2)	0.98
	O—H	2	0.958 (7)	0.964 (7)	0.994 (3)	0.005 (6)	0.43

**Table:** Non-H atoms.

Compound	〈*U*_fragHAR_/*U*_HAR_〉	〈*U_ij_*〉	〈*U_ii_*〉	*w*RMSD
GA	0.999 (9)	0.00006 (7)	0.00005 (4)	0.51
AHA	1.000 (6)	0.00005 (6)	0.00005 (7)	0.59
A_4_P_2_	0.999 (2)	0.00001 (2)	0.00001 (2)	0.24

**Table:** H atoms.

	〈*U*_fragHAR_/*U*_HAR_〉	〈*U_ij_*〉	〈*U_ii_*〉	*w*RMSD
GA	1.00 (5)	0.0007 (6)	0.0010 (10)	0.20
AHA	1.0 (3)	0.001 (2)	0.003 (5)	0.62
A_4_P_2_	1.01 (5)	0.0005 (6)	0.000 (2)	0.19
