# Supplementary material for: fragHAR: towards ab initio quantum-crystallographic X-ray structure refinement for polypeptides and proteins
Source: IUCrJ. 2020 Jan 17;7(Pt 2):158–65. doi: 10.1107/S2052252519015975 (PMC7055371; doi:10.1107/S2052252519015975)
Supplement: Supplementary file 1 [file m-07-00158-sup1.pdf]

IUCrJ

Volume 6 (2019)

Supporting information for article:

fragHAR: towards *ab initio* quantum-crystallographic X-ray structure refinement for polypeptides and proteins

Justin Bergmann, Max Davidson, Esko Oksanen, Ulf Ryde and Dylan Jayatilaka

S1. GA

In this section we display the GA dipeptide, and the fragments used.

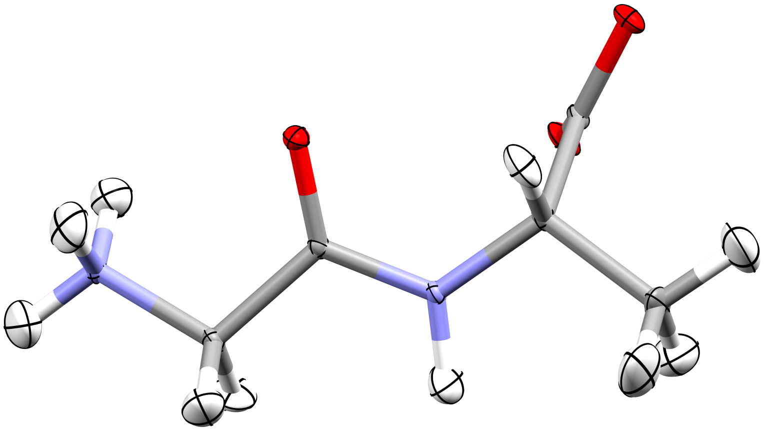

Fig. S1. Gly-Ala (GA)

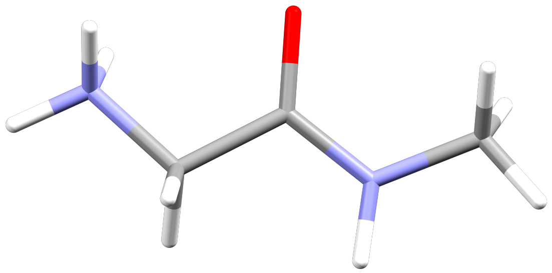

Fig. S2. First fragment of GA, GLY-1

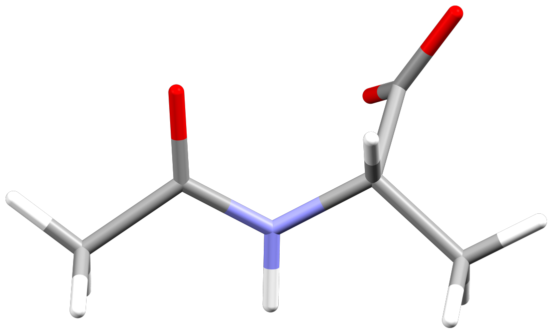

Fig. S3. Second fragment of GA, ALA-2

S1.1. Bond lengths

Figures S4–S6 are graphs of the bond lengths from fragHAR vs HAR refinements.

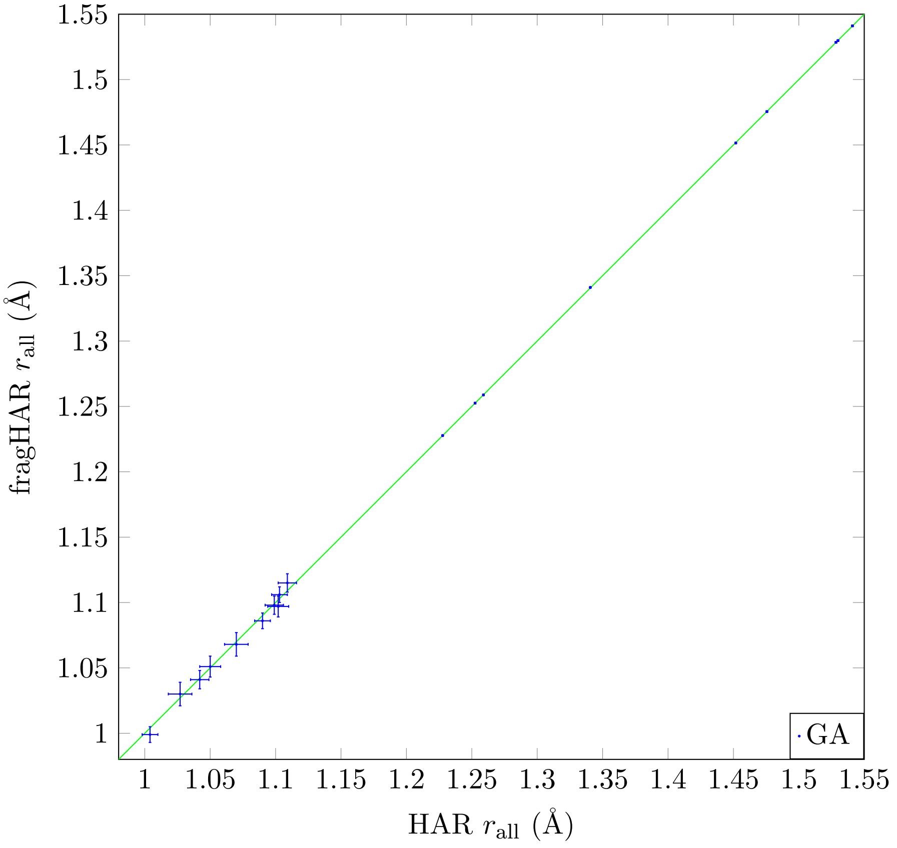

Fig. S4. Bond lengths in GA for fragHAR versus reference HAR calculations, for all atoms.

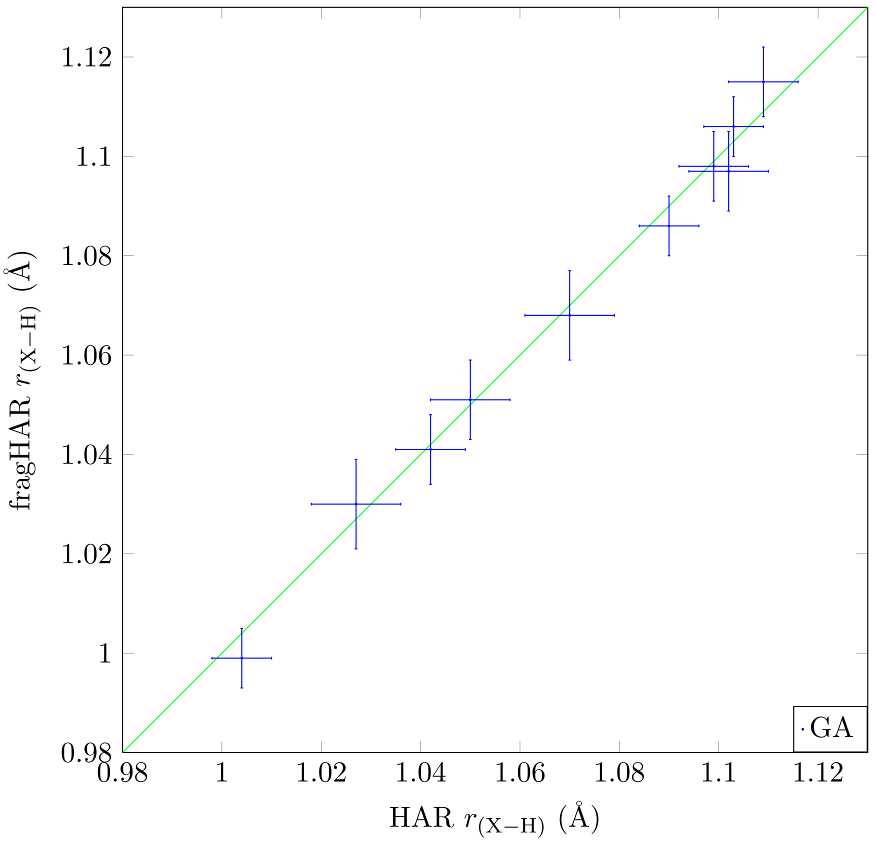

Fig. S5. X−H bond lengths in GA for fragHAR versus reference HAR calculations.

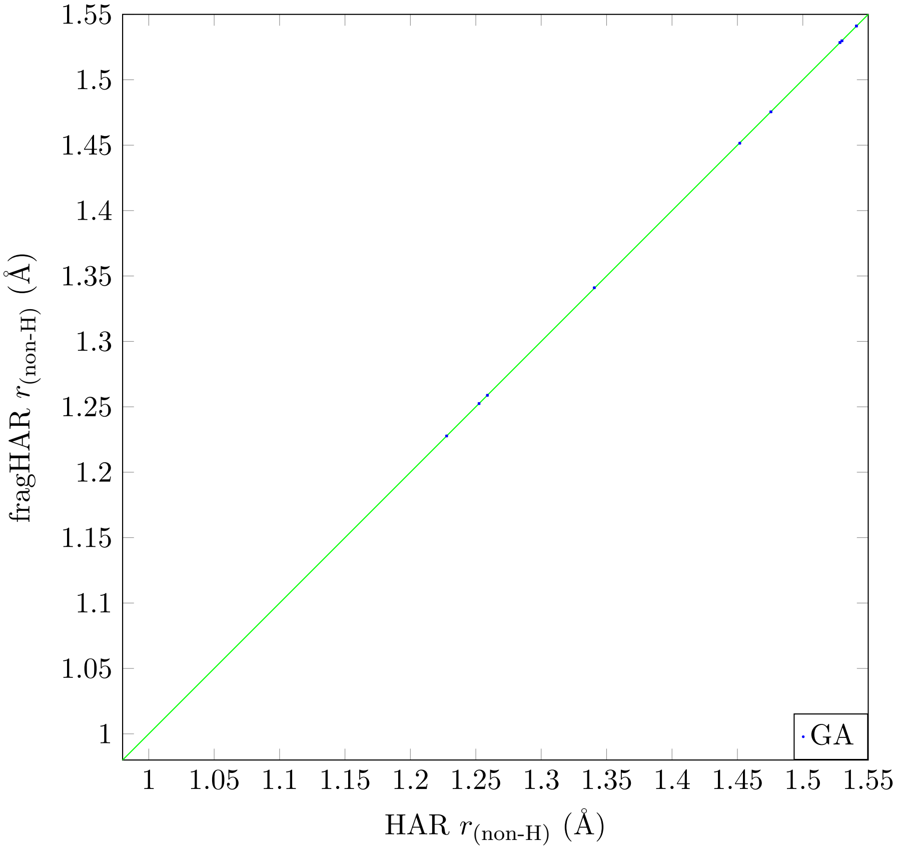

Fig. S6. Non-hydrogen bond lengths in GA, for fragHAR versus reference HAR calculations.

S1.2. ADPs

Figures S7–S9 show the atomic displaceent parameters for Gly-Ala from fragHAR plotted against HAR refinements.

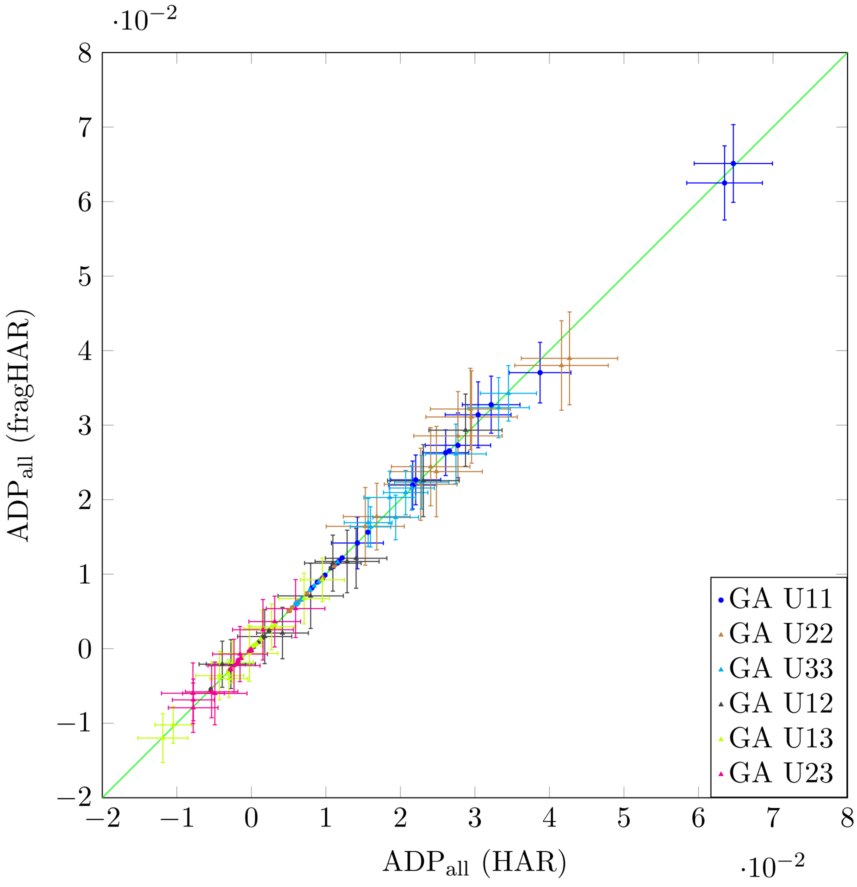

Fig. S7. Atomic displacement parameters in GA for fragHAR versus reference HAR calculations, for all atoms.

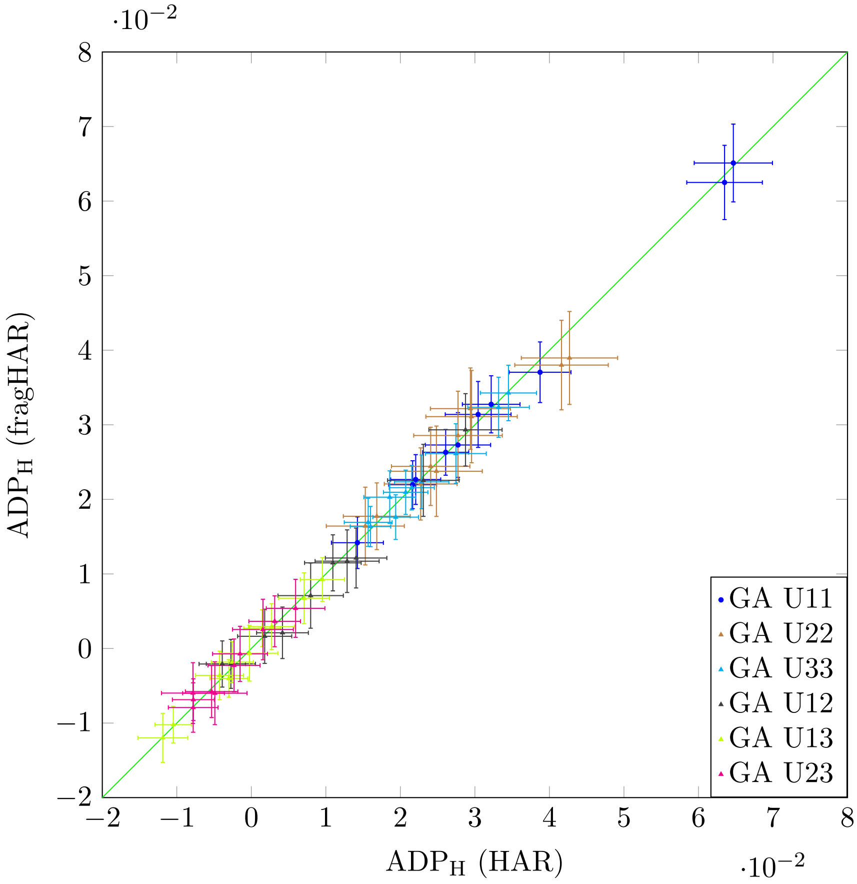

Fig. S8. Hydrogen atomic displacement parameters in GA for fragHAR versus reference HAR calculations.

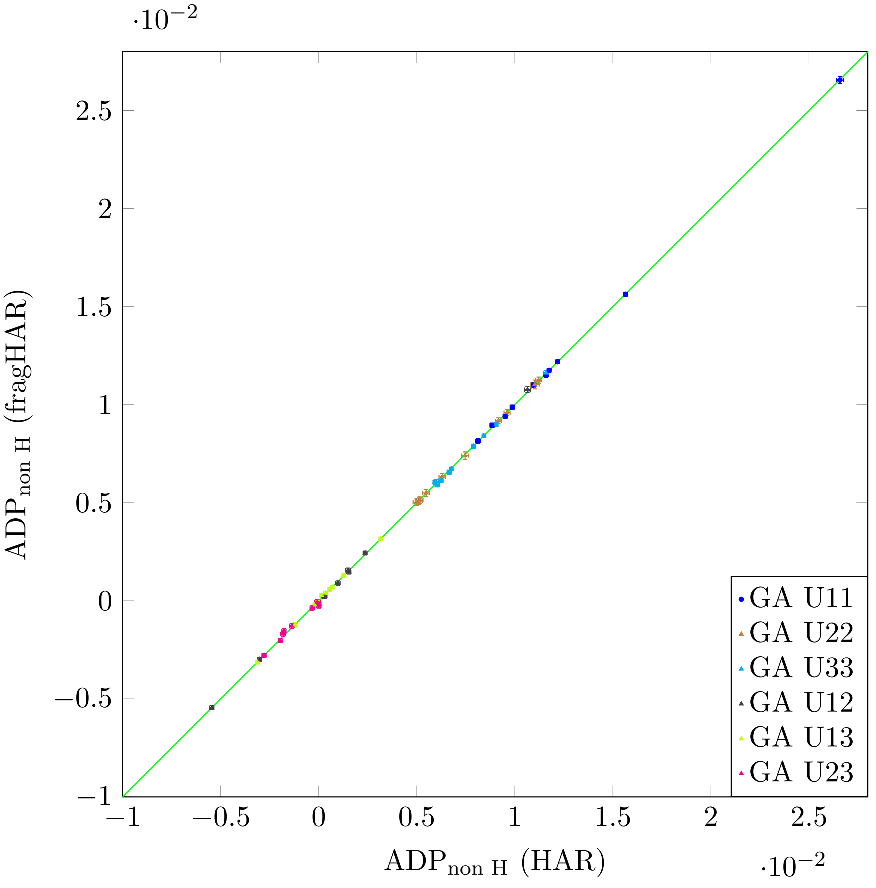

Fig. S9. Non-hydrogen atomic displacement parameters in GA for fragHAR versus reference HAR calculations.

S2. AHA

In this section we display the AHA tripeptide, and the fragments used.

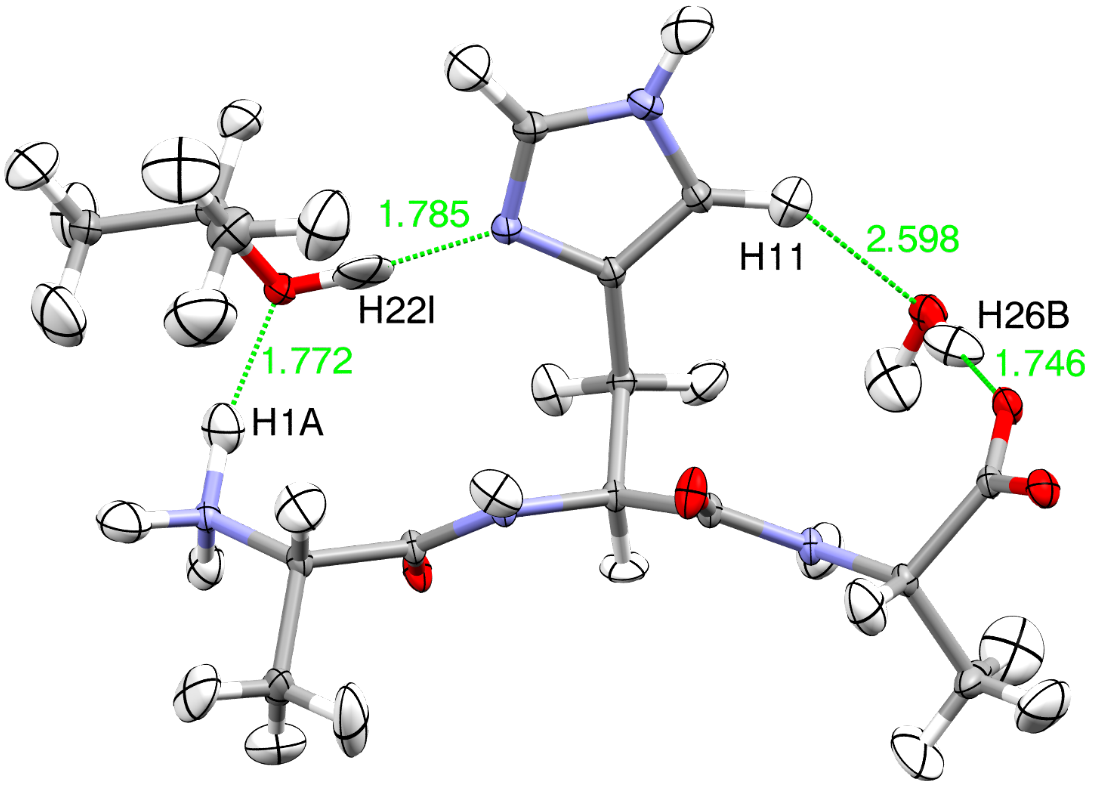

Fig. S10. A_4P_2

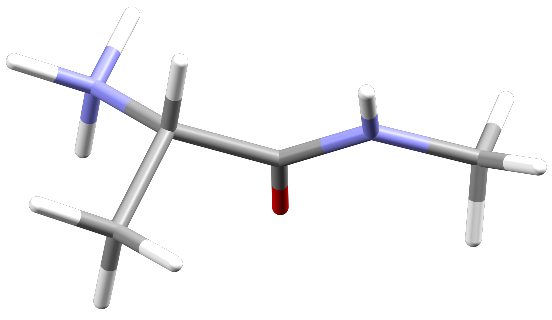

Fig. S11. The ALA-1 fragment

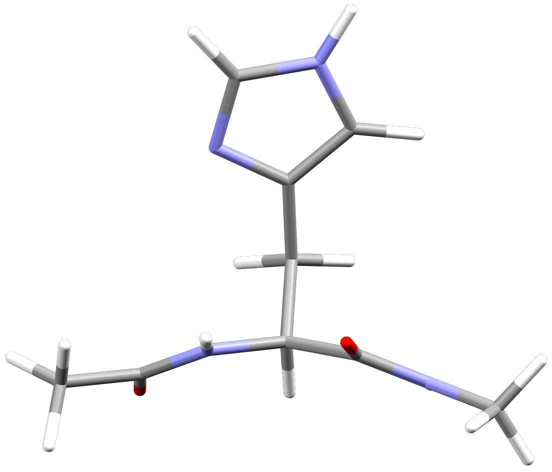

Fig. S12. The HIS-2 fragment

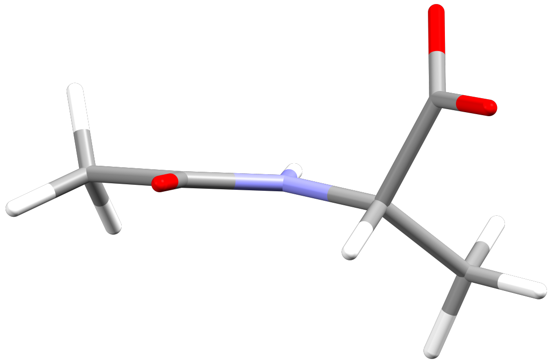

Fig. S13. The ALA-3 fragment

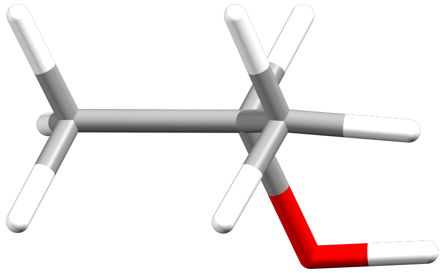

Fig. S14. The PRP-4 fragment

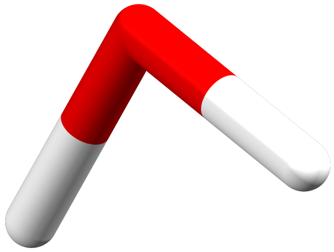

Fig. S15. The WAT-5 fragment

S3. Bond lengths

Figures S16–S18 are graphs of the bond lengths from fragHAR vs HAR refinements.

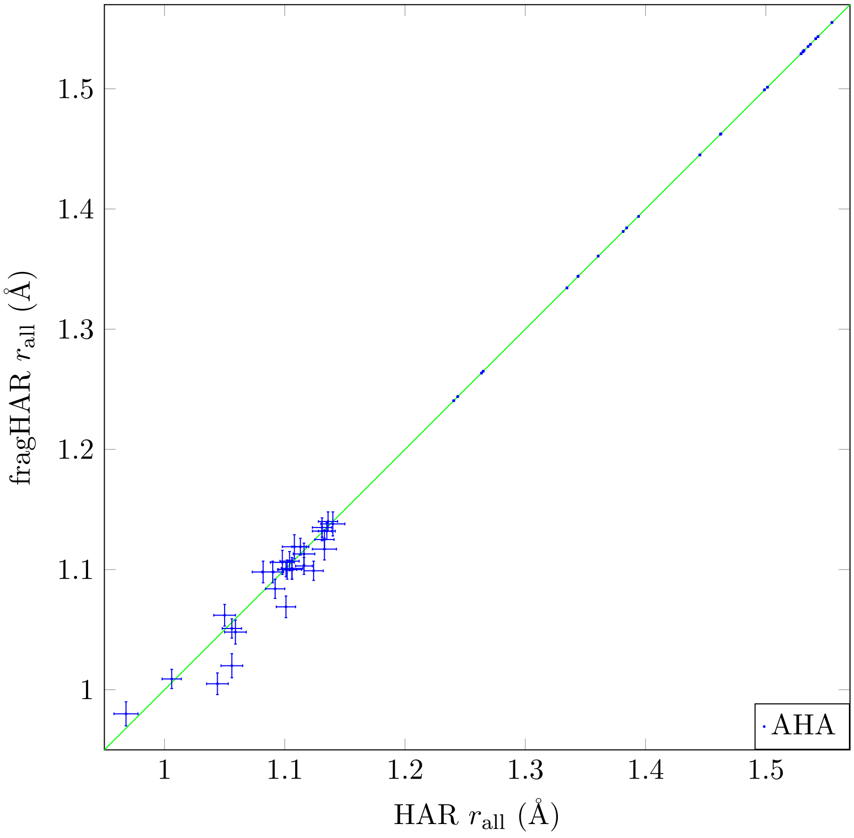

Fig. S16. Bond lengths in AHA for fragHAR versus reference HAR calculations.

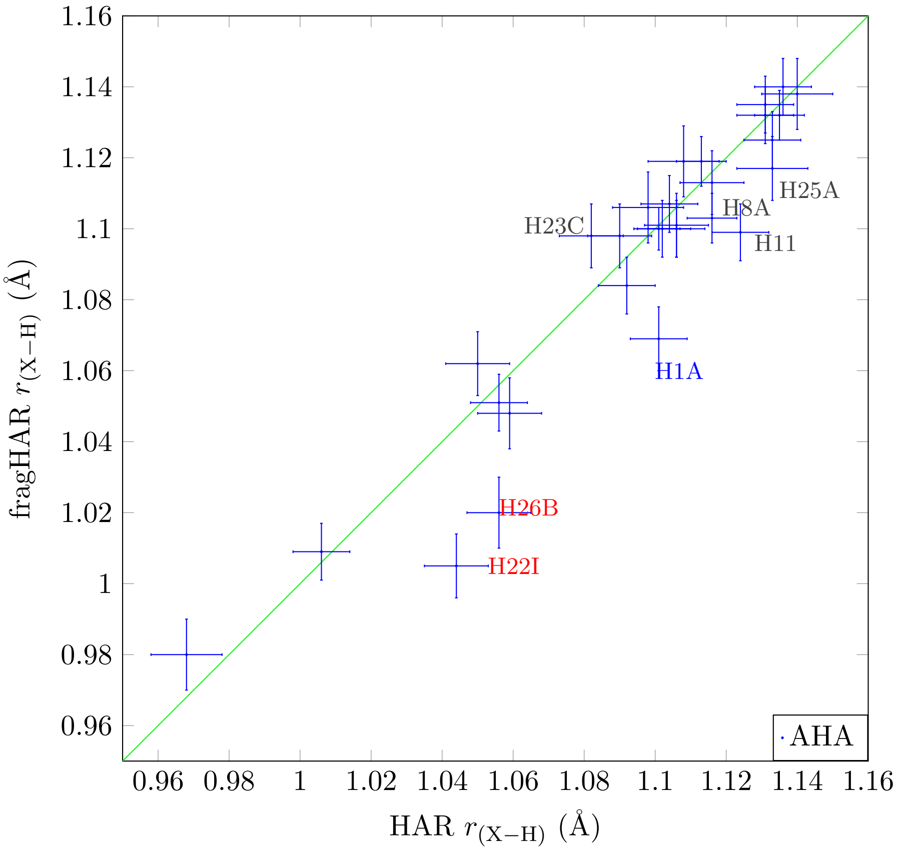

Fig. S17. X−H bond lengths in AHA for fragHAR versus reference HAR calculations.

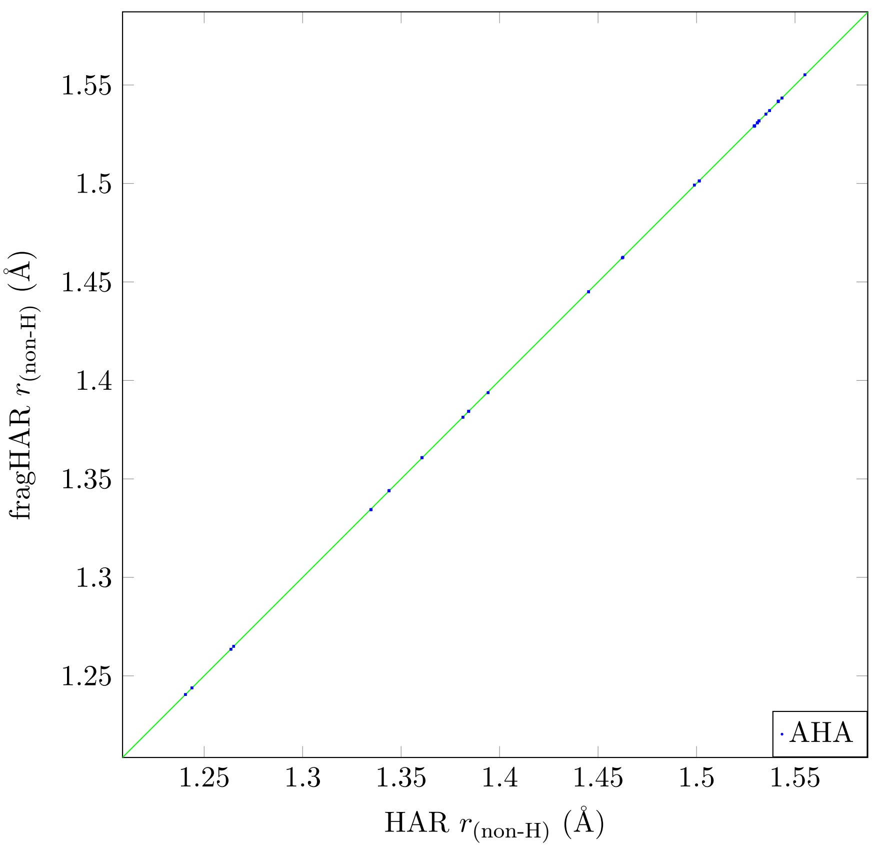

Fig. S18. Non-hydrogen bond lengths in AHA, for fragHAR versus reference HAR calculations.

S4. ADPs

Figures S19–S21 show the atomic displaceent parameters for AHA from fragHAR plotted against HAR refinements.

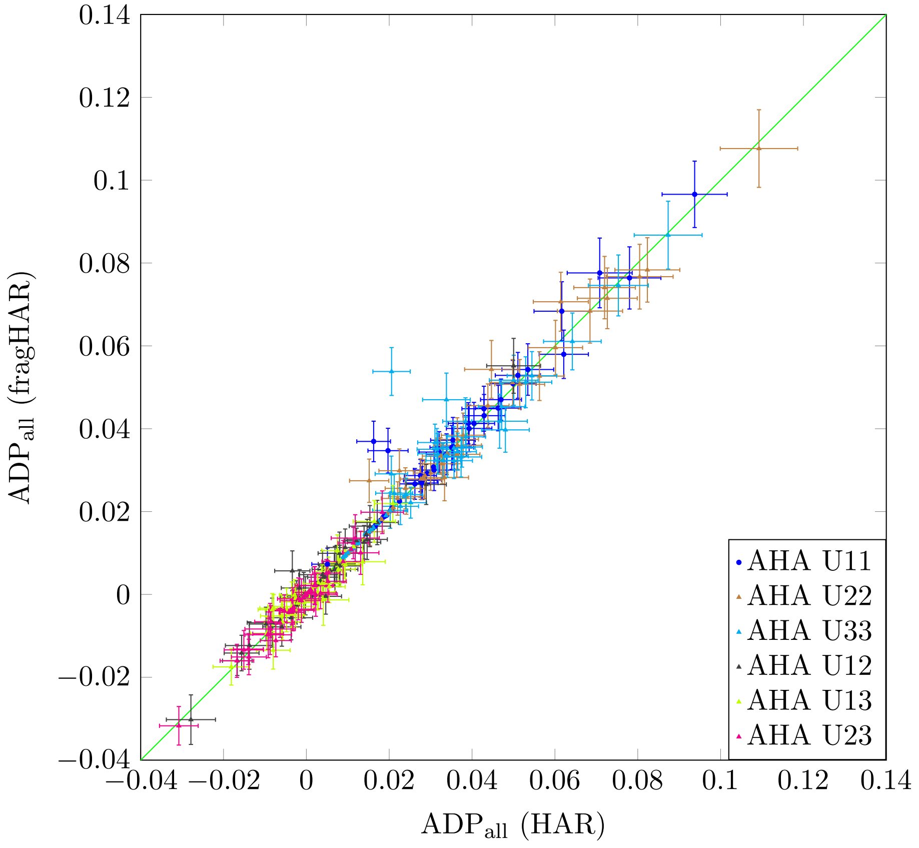

Fig. S19. Atomic displacement parameters in AHA for fragHAR versus reference HAR calculations, for all atoms.

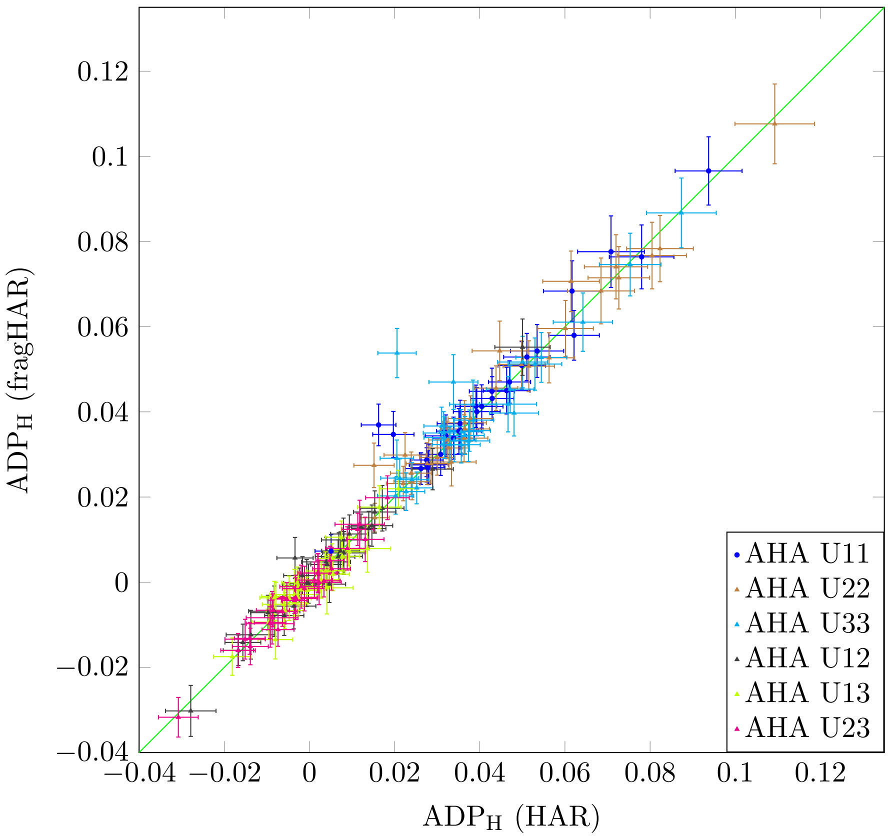

Fig. S20. Hydrogen atomic displacement parameters in AHA for fragHAR versus reference HAR calculations.

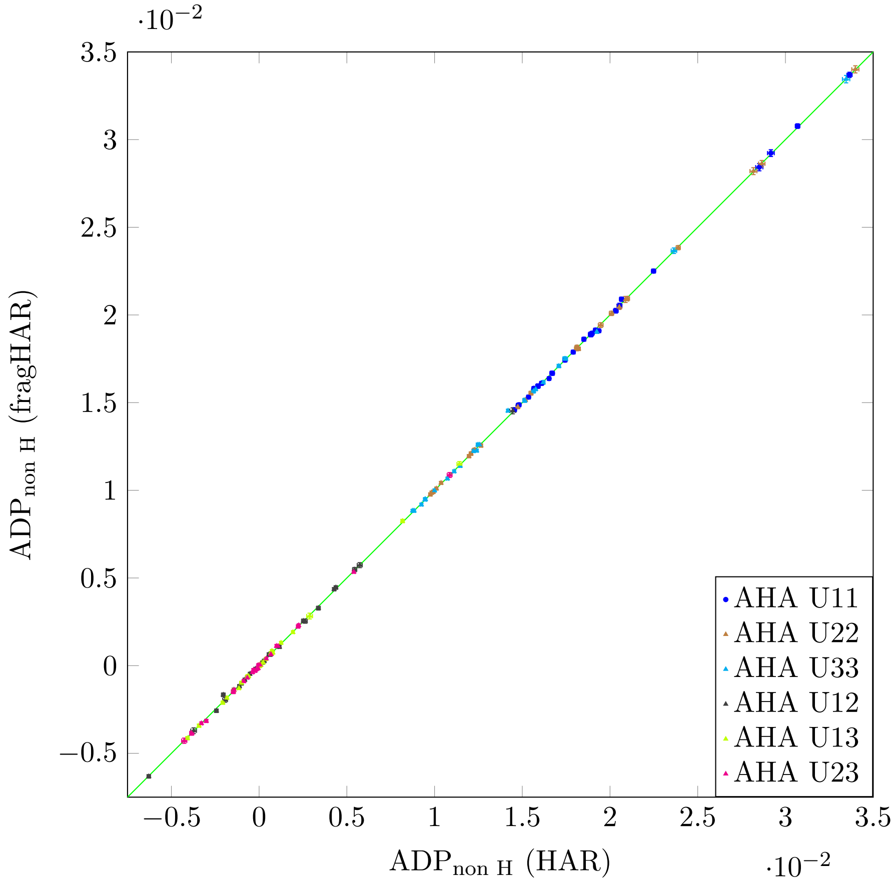

Fig. S21. Non-hydrogen atomic displacement parameters in AHA for fragHAR versus reference HAR calculations.

S5. A_4P_2

In this section we display the A_4P_2 hexapeptide, and the fragments used.

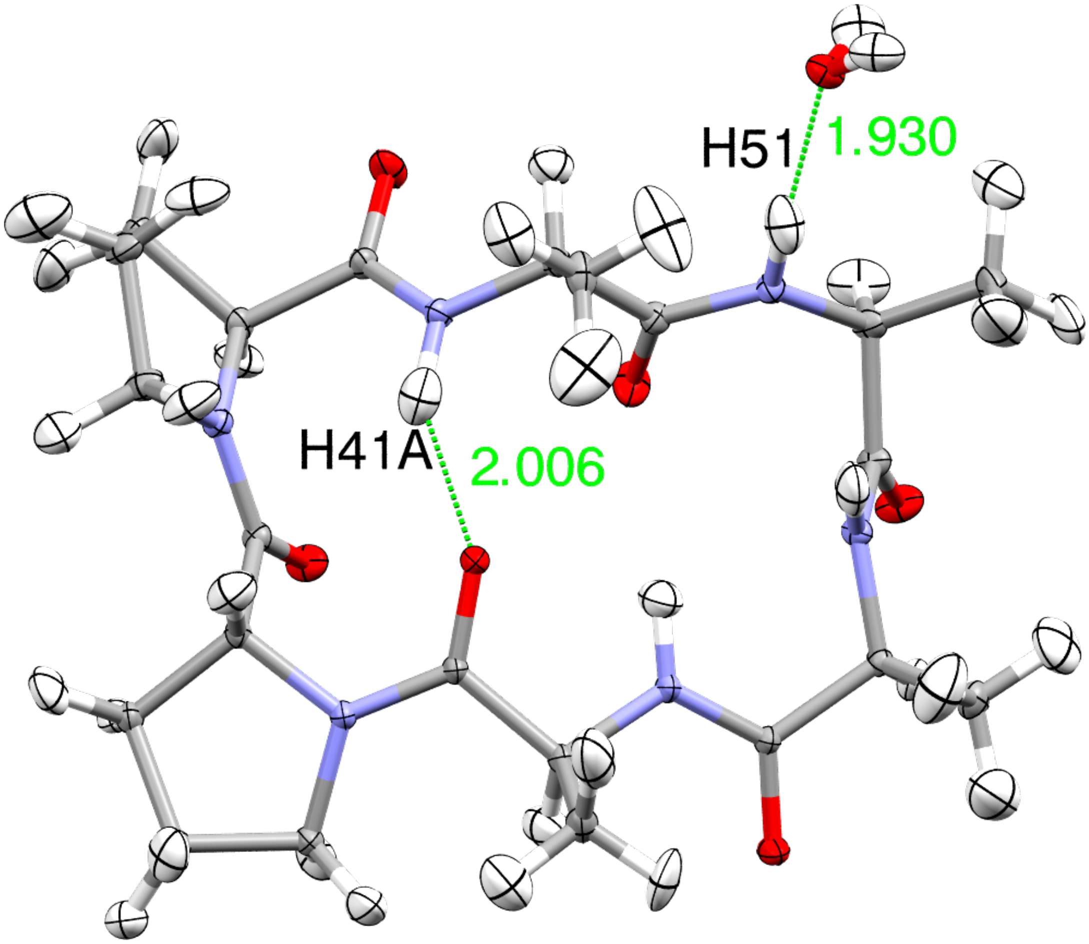

Fig. S22. A_4P_2

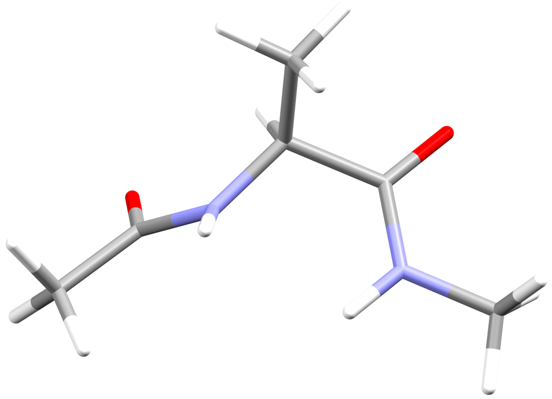

Fig. S23. The ALA-1 fragment in A_4P_2

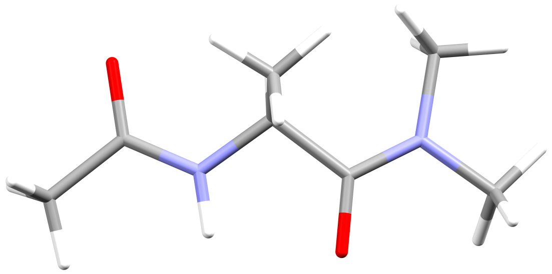

Fig. S24. The ALA-2 fragment in A_4P_2

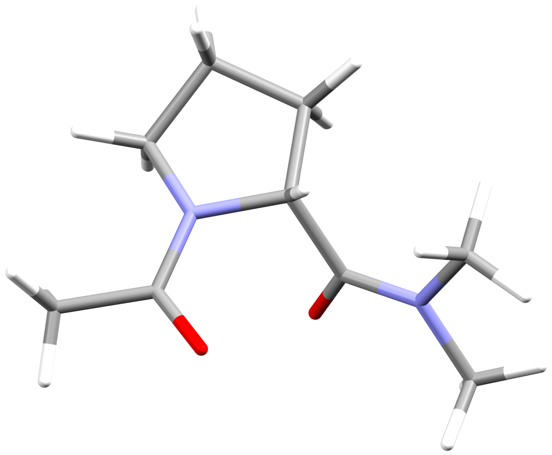

Fig. S25. The PRO-3 fragment in A_4P_2

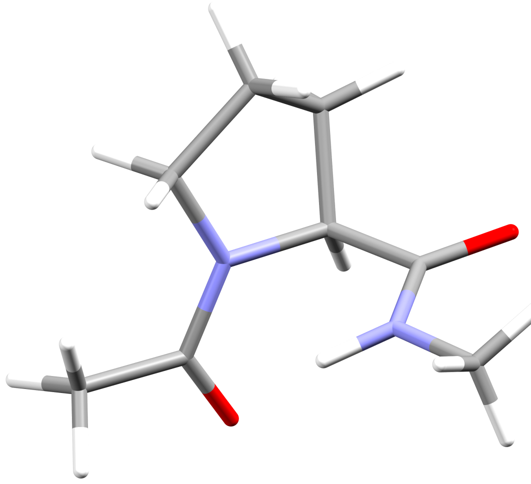

Fig. S26. The PRO-4 fragment in A_4P_2

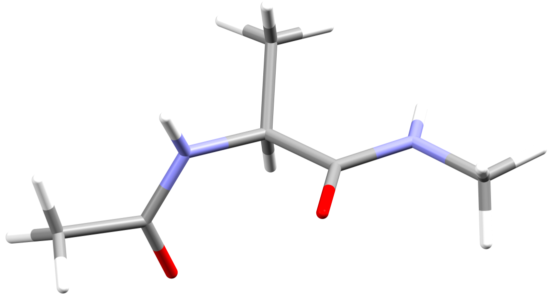

Fig. S27. The ALA-5 fragment in A_4P_2

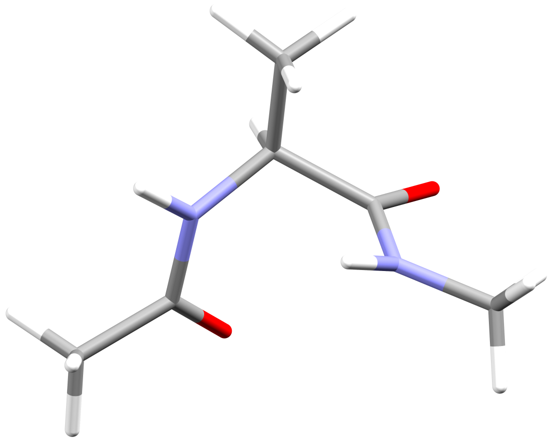

Fig. S28. The ALA-6 fragment in A_4P_2

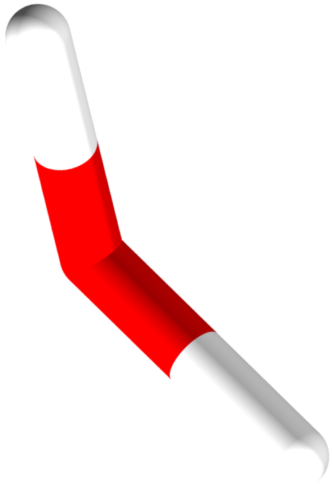

Fig. S29. The WAT-7 fragment in A_4P_2

S6. Bond lengths

Figures S30–S32 are graphs of the bond lengths from fragHAR vs HAR refinements.

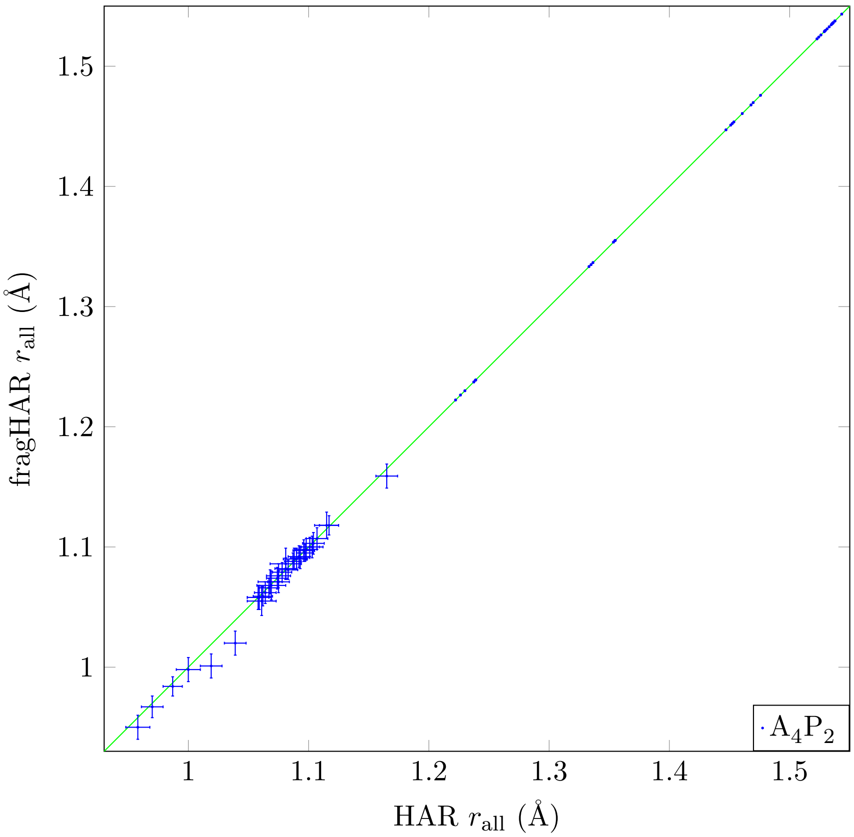

Fig. S30. Bond lengths in A_4P_2 for fragHAR versus reference HAR calculations.

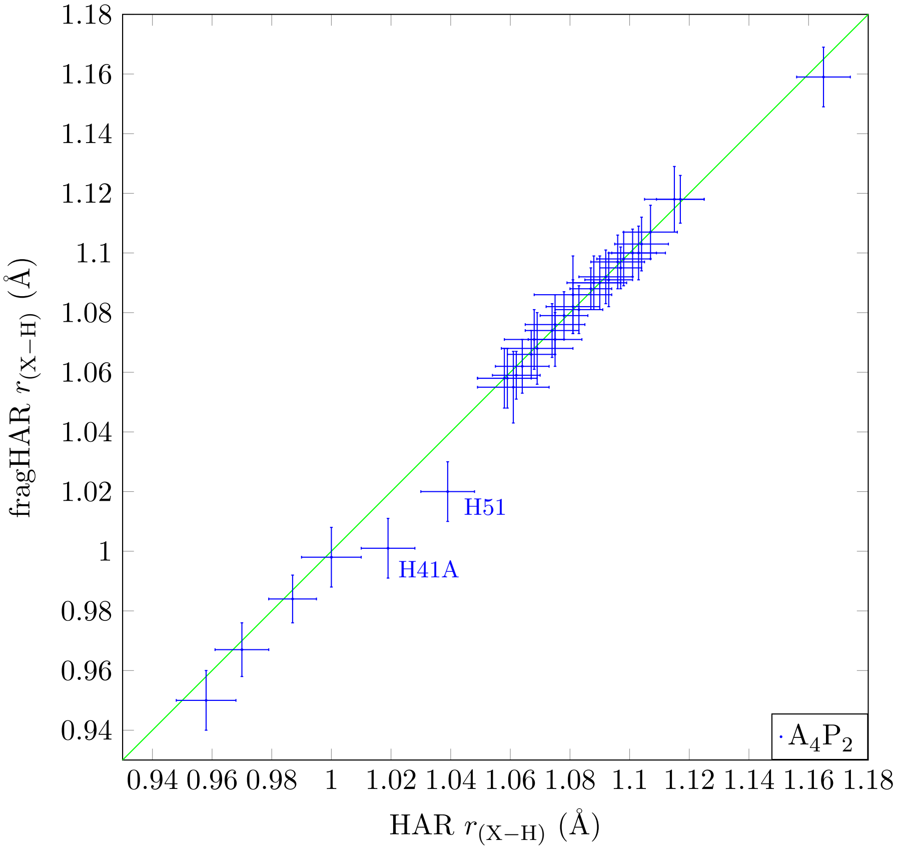

Fig. S31. X−H bond lengths in A_4P_2 for fragHAR versus reference HAR calculations.

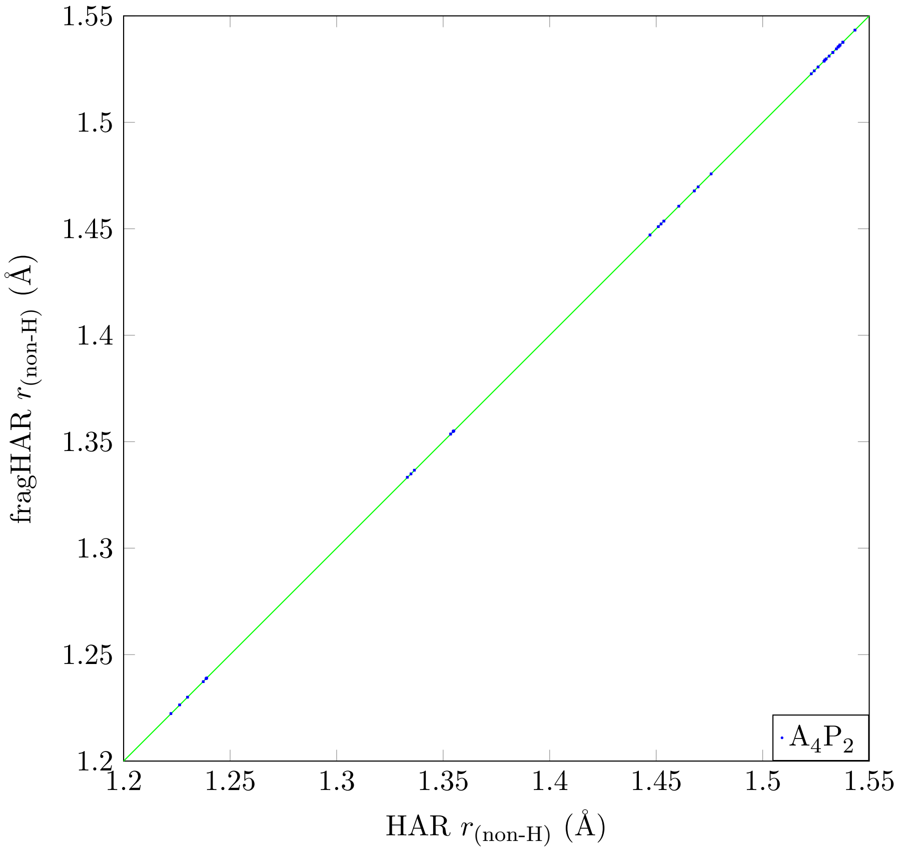

Fig. S32. Non-hydrogen bond lengths in A4P2, for fragHAR versus reference HAR calculations.

S7. ADPs

Figures S33–S35 show the atomic displacement parameters for A_4P_2 from fragHAR plotted against HAR refinements.

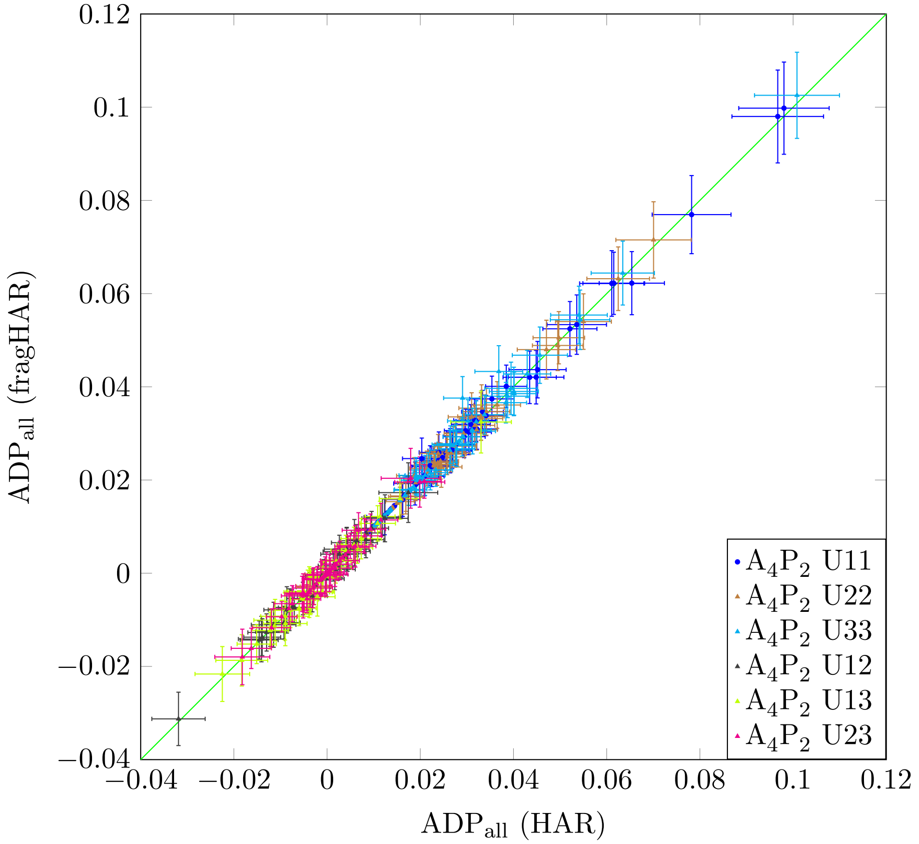

Fig. S33. Atomic displacement parameters in A_4P_2 for fragHAR versus reference HAR calculations, for all atoms.

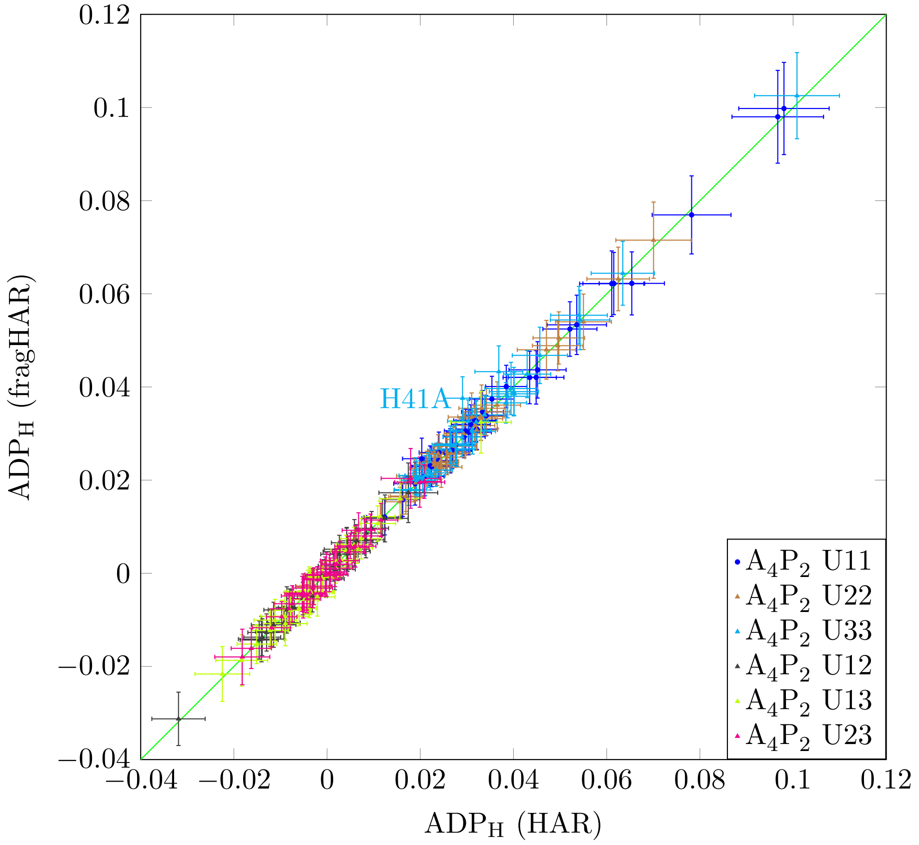

Fig. S34. Hydrogen atomic displacement parameters in A_4P_2 for fragHAR versus reference HAR claulctions.

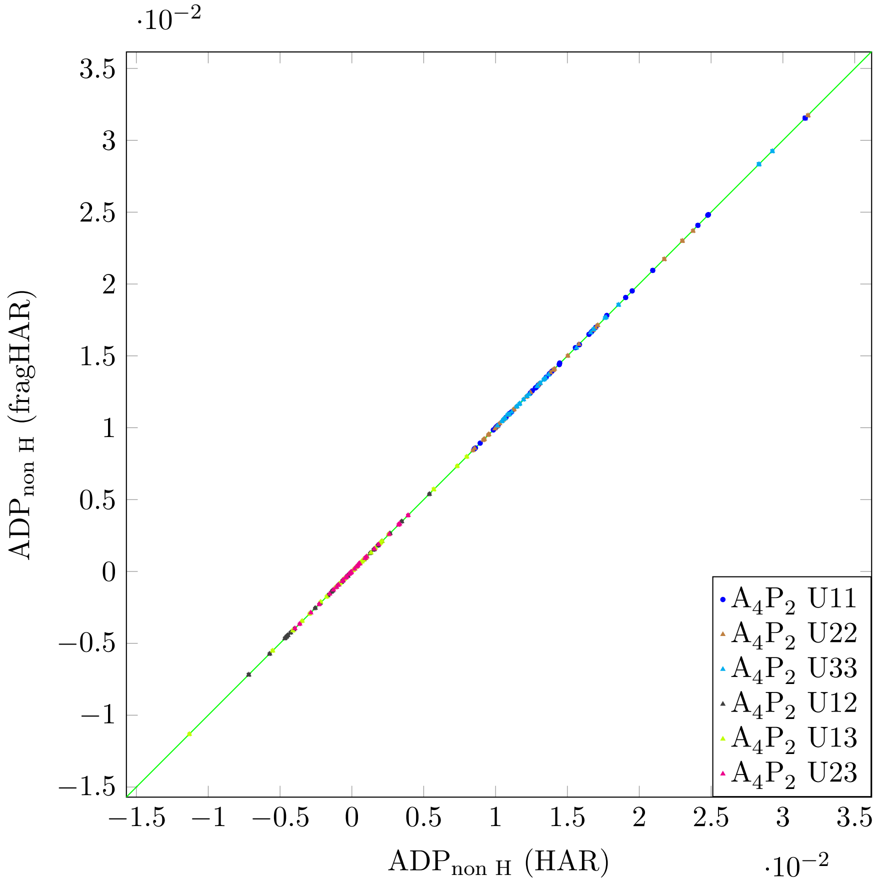

Fig. S35. Non-hydrogen atomic displacement parameters for A_4P_2 for fragHAR versus reference HAR calculations.

S8. Indicative walltime for parallel fragHAR calculations

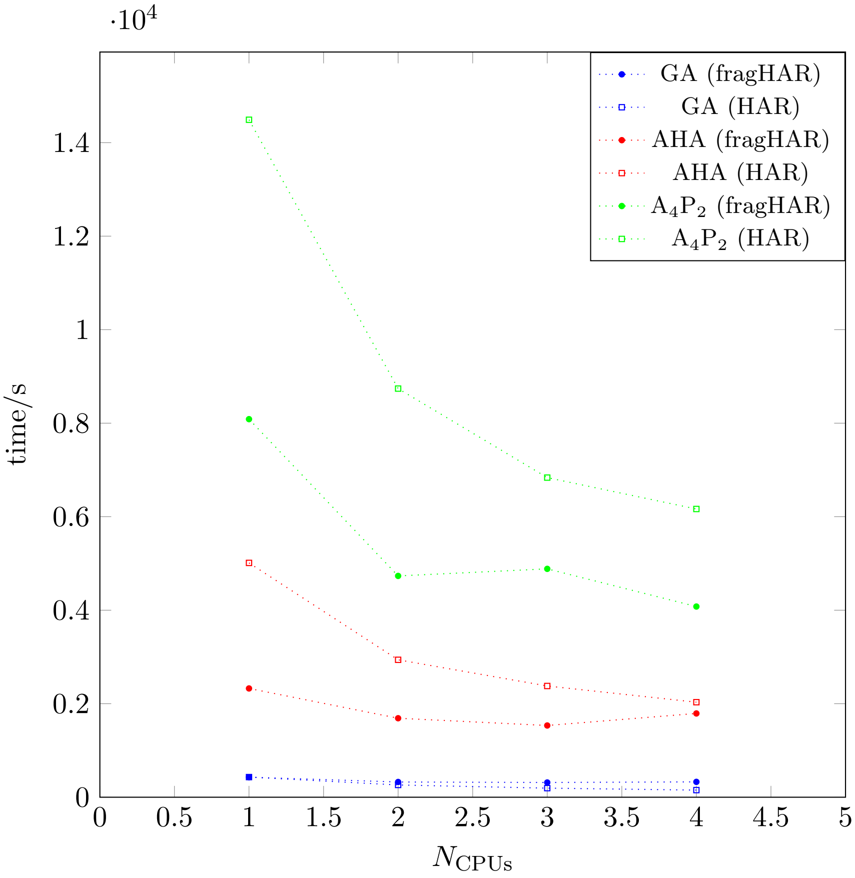

Fig. S36. Indicative walltimes for parallel fragHAR calculations, versus benchmark HAR calculations, as a function of the number of CPUs used.
